# Initial Damage and Frailty: Going to Sleep Hungry in Childhood

**DOI:** 10.1093/geroni/igaf122.2372

**Published:** 2025-12-31

**Authors:** Heng Wu, Kenneth Rockwood

**Affiliations:** Dalhousie University Department of Medicine, Halifax, Nova Scotia, Canada; Dalhousie University, Halifax, Nova Scotia, Canada

## Abstract

Frailty deficits accumulate as individuals age, whereas resilience (the ability to repair damage) and robustness (the ability to resist damage) underlie the speed of aging from one birthday to the next. Nutritional deprivation reduces the redundancies that allow robustness and resilience. In childhood, nutritional deprivation increases the burden of initial damage. Even though the functional capacity of organs in young adults is high, changes in health are often influenced by social circumstance. Here, we studied the overall impact of childhood hunger on a population’s frailty. We applied a deficit accumulation approach to construct a 47-item frailty index with domains of morbidities, cognition, lifestyle, and disabilities. We analyzed data from 10260 people aged 65+ years, participants in the Chinese Longitudinal Healthy Longevity Survey (CLHLS) 2005 cohort. They were followed in 2008, 2011, 2014, and 2018 (men=4570, age=84.6±10.6, years of education=3.5±5.6; women=6150, age=89.6±11.6, years of education=0.8±4.5). Most participants (73.0%) responded that they had slept hungry as children. A regression model using Generalized Estimating Equations showed that all predictors (sex, age, education, hungry in childhood) significantly affected the frailty index (p-value < 0.001). We found that children who had not experienced hunger had fewer health deficits (beta coefficient for “not hungry” = -0.010 (95% CI=-0.02∼-0.01) versus “hungry”, adjusted for age, sex, and education. Reflecting better ability to repair, as older adults, they experienced less smooth transitions in aging. In short, children who did not experience hunger on average have a slower speed of aging, with less frailty and greater resilience in old age.

